# Therapeutic Monoclonal Antibodies and Emergence of Their Biosimilars

**Published:** 2018

**Authors:** Mahmood Jeddi-Tehrani

**Affiliations:** Department of Hybridoma, Monoclonal Antibody Research Center, Avicenna Research Institute, ACECR, Tehran, Iran

Antibodies are proteins of the immune system that are produced by B-lymphocytes. These proteins exert their effects by recognizing and binding to their targets (antigens). A monoclonal antibody (mAb) is originally produced by a single B-cell. Production of mAbs was first introduced in 1975 using cell fusion technique and hybridoma cell production. A Hybridoma is formed by fusion of an antibody producing B-lymphocyte and a myeloma cell line. These cells have two main characteristics, production of uniform, monospecific antibodies (mAbs) that originate from the B-cell and immortality that comes from the myeloma cell line. A hybridoma cell line is thus acting like a biological factory that produces and secretes mAbs into the cell culture medium. Most of the mAbs have been produced in mice and are thus proteins of murine origin. mAbs were later found to be able to bind biological targets like tumor antigens, molecules involved in autoimmune and infectious disease-related molecules, *etc*. This led to emergence of therapeutic monoclonal antibodies. However, since the first mAbs were murine proteins (OKT3 was injected to kidney transplant patients to prevent graft rejection), their repeated administration raised Human Anti Mouse Antibody (HAMA) in the patients that resulted in neutralization of the injected mAb (OKT3). The solution was to genetically change the murince antibodies to human antibodies. In this regard chimeric (%80–%90 human), humanized (≥%90 human) and fully human (%100 human) therapeutic mAbs were produced. At present more than 50 therapeutic mAbs are on the market with more than 120 billion USD global market share. These mAbs have shown very good therapeutic effect in treatment of cancers, autoimmune and infectious diseases. However, these drugs are very expensive and thus their patient accessibility is limited. Although, they are protected by patents, some patents have already expired and some are close to expiration dates. Here, biosimilar versions of these drugs have started to immerge. Biosimilars are defined as biological drugs that are highly similar but not identical to the biological reference (original or originator) drug. The approval of biosimilars to enter the biopharmaceutical market is governed by the regulatory bodies in different countries. This happens under strict and comprehensive comparability exercise. The biosimilarity determination procedure is planned to ensure that the difference between the originator and the biosimilar drug is not clinically significant. The assessment includes immunochemical and physicochemical properties, biological activity, structural similarity, purity, contamination with impurities like host cell protein and DNA. In addition, in vivo pharmacology studies like pharmacokinetic and pharmaco-dynamic characteristics as well as, efficacy, safety and tolerability must be determined and approved. Moreover, after market analyses like pharmaco-epidemiological studies should also be done.

These procedures are also necessary to be performed to assure the prescribing physicians to suggest them to their patients. Thus, the biosimilars, due to their lower production costs, are expected to introduce huge amounts of cost savings to healthcare systems and more importantly, increase affordability/accessibility of biological treatment.

